# Evaluation of Acellular Intact Fish Skin Grafts for Treating Acute and Chronic Wounds

**DOI:** 10.1111/iwj.70931

**Published:** 2026-04-27

**Authors:** Christopher Böddeker, Sarah Biermann, Theresa Maria Jansen, Bernhard Homey, Norman‐Philipp Hoff

**Affiliations:** ^1^ Department of Dermatology University Hospital Duesseldorf, Medical Faculty, Heinrich‐Heine‐University Duesseldorf Duesseldorf Germany

**Keywords:** acellular dermal graft, chronic wounds, fish skin, fish skin transplantation, hard‐to‐heal wounds, wound healing

## Abstract

Acute, chronic, and hard‐to‐heal wounds pose a growing challenge due to reduced patient quality‐of‐life, higher cost and increased hospital admissions. Although there is no gold standard for wound care, there is a trend toward simple, outpatient‐compatible treatment options. Acellular dermal fish skin derived from *Gadus morhua* (Atlantic cod) is a promising alternative. This retrospective study evaluated the surgical procedures and outcomes associated with fish‐skin graft transplantation for difficult‐to‐treat wounds. Forty‐four patients (33 male and 11 female; mean age: 72.4 years; mean wound area: 84.5 cm^2^) were treated with acellular fish skin graft. Wound healing was assessed by photographic documentation, confocal microscopy and clinical evaluation. Patient satisfaction was assessed using a structured questionnaire. A positive effect on wound healing, defined as a reduction in wound size, was observed in 88.6% of cases, with complete wound closure in 45.45% of cases. Most patients reported that the procedure was neither painful nor time‐intensive; 95% stated that they would opt for treatment again. Fish skin grafting has also shown beneficial outcomes in complex cases, such as in wounds secondary to pyoderma gangrenosum and wounds with exposed bone. Fish skin graft transplantation represents a safe, well‐tolerated and effective option for managing chronic wounds as well as challenging postoperative wounds, such as those at anatomically challenging sites.

## Highlights

- High healing response in real‐world complex wounds.
- Effective even in anatomically difficult and high‐risk wounds.
- Minimally invasive, outpatient‐compatible procedure.
- Excellent safety profile with no major complications.
- Strong patient acceptance (95% would repeat treatment).
- Potential to avoid more invasive reconstructive procedures.
- Evidence for use in rare inflammatory ulcerative conditions.

## 1 Introduction

In dermatology, we are increasingly encountering patients with complex dermatological conditions, including advanced‐stage skin tumours, chronic wounds and challenging (post‐) surgical skin defects [1]. These clinical challenges are becoming more prevalent and severe, often involving significant tissue loss, increased risk of infection and delayed wound healing. A considerable proportion of these patients are elderly and suffer from multiple chronic comorbidities, such as diabetes mellitus, peripheral arterial disease or immunosuppression, which further compromise the physiological wound healing process and increase the likelihood of complications [2]. Chronic non‐healing wounds constitute an increasingly significant healthcare challenge within the aging populations of industrialized nations, often resulting from multifactorial etiologies [3]. These wounds are typically defined as those that fail to exhibit substantial healing progress within a two‐month period, thereby imposing a considerable burden on patients, physicians, and healthcare systems. In the field of dermatosurgery, many acute wounds are made that cannot be closed immediately following oncological dermatosurgical procedures, especially in elderly patients [4]. In Germany, it was estimated that, in 2017, 200 000 individuals were diagnosed with keratinocyte cancer and 22 890 with malignant melanoma, and these numbers are continuously increasing [5, 6]. Complete excision is the preferred treatment for skin tumours. However, depending on the location, histology and size of the defect, primary wound closure may not always be feasible. In such cases, secondary wound healing is used, as in the management of chronic wounds. If necessary, defect coverage is accomplished through the application of a skin graft. Concurrently, we face escalating pressures within the healthcare system, including limited financial and personnel resources as well as time constraints that impact the delivery of individualized patient care. These systemic challenges necessitate the adoption of novel, evidence‐based and cost‐effective therapeutic strategies by dermatologists and dermatologic surgeons. Innovative treatments are essential for improving wound healing outcomes, reducing hospital stays and enhancing patient quality of life. Optimal wound care requires a multidisciplinary approach that combines targeted treatment of the underlying cause with appropriate local wound management [7]. Therapeutic decisions are influenced by various wound‐related factors, including wound size, location, exudate level and extent of microbial colonization [8].

Intact fish skin graft (IFSG), composed of decellularized fish skin from Atlantic cod, is increasingly used in the field of dermatosurgery [9]. IFSG has demonstrated promising clinical efficacy in the management of both chronic and thermal wounds [10, 11, 12]. In addition to its structural compatibility with the human dermis, as evidenced by electron microscopy, IFSG possesses a high concentration of omega‐3 fatty acids, molecules that have been shown to have antimicrobial, antiviral and anti‐inflammatory properties. IFSG has been shown to facilitate cellular migration, proliferation and ingrowth, thereby supporting the wound‐healing process [13, 14, 15, 16].

Owing to its distinctive properties, IFSG has emerged as a promising biomaterial for the treatment of various wounds. The application of IFSG in dermatological wound care remains underexplored, and its potential to improve wound‐healing outcomes in the dermatosurgical field is largely uncertain. In the present study, we describe patients with chronic or complex cutaneous wounds that have been treated with IFSG in accordance with the indications of the product in recent years. In most of these cases, significant progression in wound healing was achieved, with many patients progressing to complete wound closure. This retrospective study aimed to evaluate the clinical efficacy of IFSG as well as patient satisfaction in those treated with IFSG by analysing clinical data extracted from our institutional documentation system.

## 2 Material and Methods

### 2.1 Data Source

The data analysed in this study were obtained from our institutional database, covering the period from 2020 to 2025. Relevant cases were identified by correlating the ICD codes assigned within the Department of Dermatology with the OPS procedure code for ‘temporary soft‐tissue coverage’ (5–916). Furthermore, we included the subcodes ‘Xenogeneic skin substitute, small’ (5–916.1) and ‘Xenogeneic skin substitute, large’ (5–916.6). In total, we identified 2421 cases at the University Hospital, coded under OPS 5–916. Among these, 50 cases involved the clinical application of IFSG within the Department of Dermatology. Of these, 44 had complete clinical documentation and were included in the final analysis.

Of the 50 identified cases, 6 patients were excluded due to missing follow‐up data. These patients could not be reached after transplantation and therefore lacked post‐interventional clinical assessment, including evaluation of wound healing and patient‐reported outcomes.

### 2.2 Study Design

Between May 2020 and February 2025, 44 patients with complex wounds of various etiologies underwent IFSG transplantation at the Department of Dermatology, UKD. These wounds included complex acute wounds, such as surgical wounds following the excision of keratinocyte cancer or melanoma, and chronic wounds such as vasculitic ulcers and those associated with pyoderma gangrenosum and chronic wound healing disorders. We conducted a retrospective assessment of transplantation outcomes using our clinical patient management system documentation (Medico), photographic records, confocal microscopy images, and distributed patient questionnaires based on the Wound‐QoL by Augustin et al. 2017 and Blome et al. 2014 [17, 18].

This retrospective study was conducted in accordance with the Declaration of Helsinki and received approval from the Ethics Committee.

### 2.3 Surgical Procedure

**FIGURE 1:**
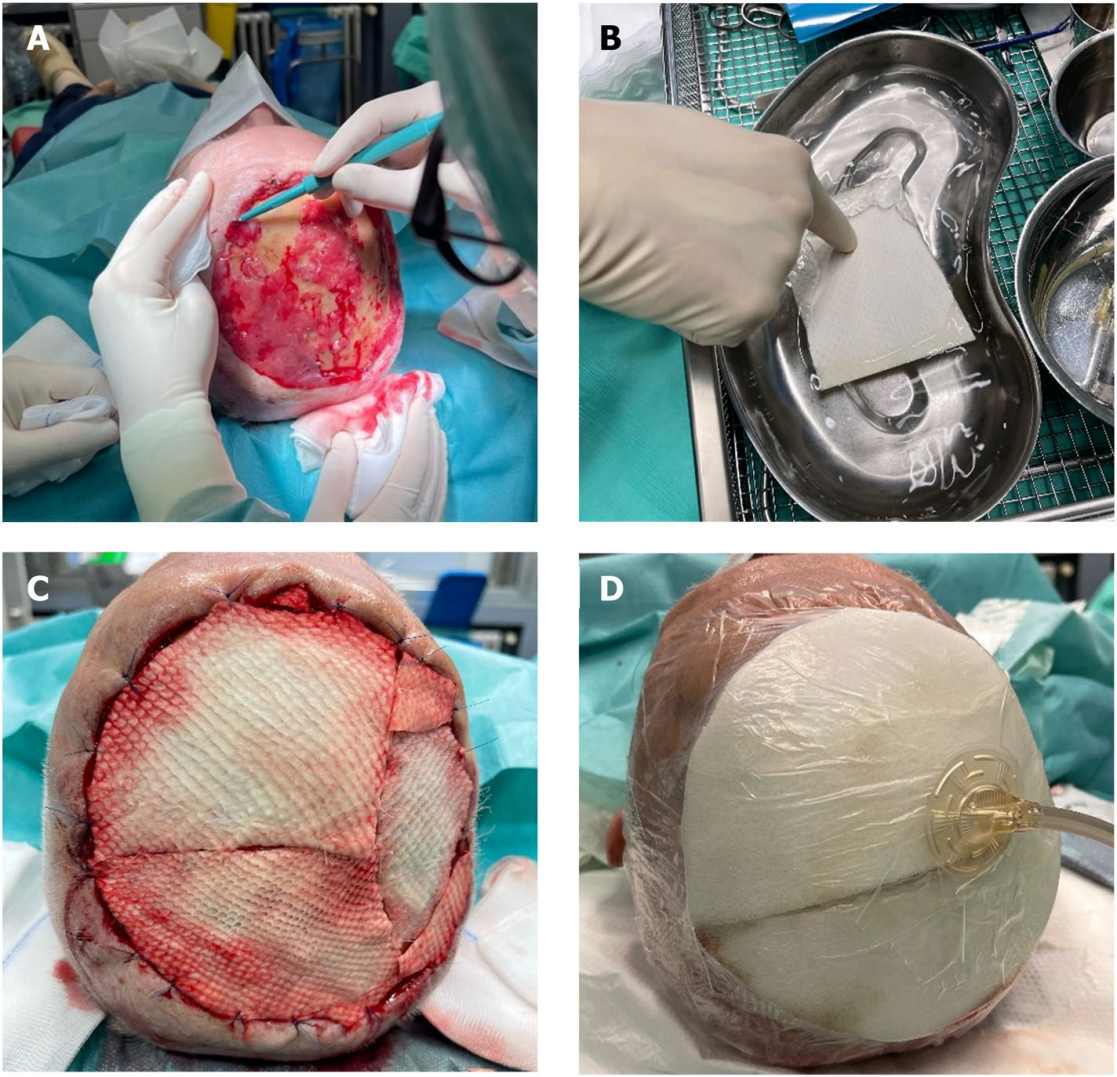
Prior to the transplantation procedure, the wound edges and bed underwent debridement (A). The acellular dermal graft (B) was subsequently immersed in 0.9% saline solution for a duration of 10–15 min and then sutured to the wound edges using resolving sutures (C). Finally, the transplantation site may be covered with either a gauze bandage utilizing non‐adherent wound dressings or a negative pressure wound therapy (NPWT) dressing, maintained under continuous suction at 75 mmHg (D).

As per the current regulatory framework in Germany, a single supplier is authorized to produce and distribute IFSG. All the participants in this study received treatment exclusively with Kerecis IFSG (Kerecis Deutschland GmbH, Am Neumarkt 42, 22 041 Hamburg, Deutschland). This treatment was administered to patients who exhibited no signs of healing despite receiving standard‐of‐care, stage‐appropriate modern wound therapy, as well as to those with postoperative wounds with a poor healing prognosis due to being in anatomically critical regions such as areas over tendons, bony prominences or being in areas previously subjected to radiotherapy. All surgical procedures were conducted under local anaesthesia using articaine 1% with epinephrine as part of routine clinical care within the dermatologic surgery unit (Figure 1). IFSG was used according to the manufacturer's guidelines. Prior to graft placement, the wound edges were debrided using a curette until pinpoint bleeding was observed, thus ensuring a viable wound bed. Concurrently, the IFSG was rehydrated in normal saline (NaCl 0.9%) for 10 min. The IFSG was then sutured to the wound margins using an absorbable suture material. Subsequently, small stab incisions were made centrally in the graft using a No. 11 scalpel blade to facilitate exudate drainage and tissue integration. Where feasible, the treated wounds were covered with a negative pressure wound therapy (NPWT) system (75 mmHg, white foam dressing) for a duration of 5 days. At our institution, NPWT is generally not applied to anatomical sites such as the fingers, toes, midface, ears and nose, primarily due to challenges in ensuring a stable dressing seal and maintaining continuous negative pressure.

In anatomical locations where NPWT was not applicable, a tie‐over bolster dressing was applied and maintained for the same period.

### 2.4 Study Variables and Outcomes

All patients received postoperative care following our institutional standard protocols, either through the outpatient dermatologic surgery unit or at the interdisciplinary University Wound Center (UWC). Follow‐up care involved detailed wound documentation, with a systematic evaluation of wound healing progress, overall clinical outcomes, and patient‐reported experiences using a modified version of the Wound‐QoL questionnaire in 20 of 44 cases (see Supporting Informations). The Wound‐QoL questionnaire is a validated instrument designed to assess quality of life in patients with chronic wounds and comprises three domains: Everyday life, body and psychological well‐being, using a 5‐point Likert scale ranging from 0 (‘not at all’) to 4 (‘very much’).

For the purpose of this study, a modified version of the Wound‐QoL questionnaire was used to reflect the specific clinical context following IFSG transplantation. The adapted version retained the original response scale but included minor modifications in item wording and selection. Certain items of the original questionnaire (e.g., odour‐related aspects) were not included, while other items were adapted to better reflect postoperative wound conditions. In addition, specific items were included to capture perioperative and treatment‐related aspects, such as pain during transplantation, wound healing status after the procedure, and the patient's willingness to undergo IFSG transplantation again. We have attached the original Wound‐QoL questionnaire from our clinical routine in Germany, along with a 1:1 translation (see Supporting Informations).

Due to the retrospective design and incomplete availability of questionnaire data (*n* = 20), no overall score or domain scores were calculated. Instead, responses were analyzed descriptively at the item level.

Missing data were not imputed; analyses were based on available responses only. Any adverse events, complications or infections were recorded during the follow‐up. Wound dressings were replaced every 2–3 days using non‐adhesive wound dressings and sterile compresses, in line with the protocol for wound care after NPWT or tie‐over bolster dressing.

Clinical evaluations, along with photographic and, in some cases, confocal microscopy documentation, were conducted at various intervals during dressing changes. Wound size was assessed based on clinical documentation and photographic records. When available, wound dimensions were extracted from clinical records; otherwise, measurements were estimated from photographic documentation. Wound area was approximated using length multiplied by width. For this retrospective study, the researchers independently defined granulation, healing and non‐healing using clinical and photographic documentation of the study participants. Complete wound healing was defined as full epithelialization of the wound without signs of secretion or the need for further wound care. Partial healing was defined as a clinically relevant reduction in wound size and/or the presence of stable granulation tissue. Patients were classified as responders if either complete or partial healing was observed, whereas non‐responders showed no clinically meaningful improvement.

Outcome assessment was performed by non‐blinded investigators, which may introduce observer bias. Information on patient follow‐up, satisfaction and interventions aimed at achieving aesthetic and functional enhancement was directly extracted from patient records.

### 2.5 Statistical Analysis

Continuous data were presented as means along with ranges or standard deviations, while categorical data were expressed as counts and percentages of the total. All data were analyzed using Microsoft Excel (Microsoft Corporation, USA). Depending on the data type, the mean values with standard deviations (SDs) or medians with ranges were calculated.

## 3 Results

### 3.1 Patient Characteristics

**FIGURE 2:**
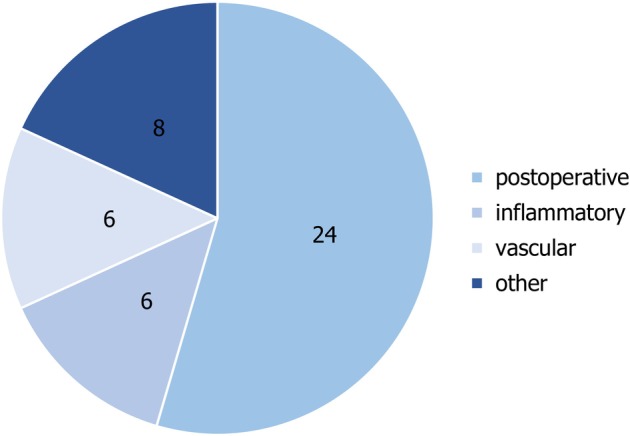
Distribution of wound etiologies observed in the study population. The chart shows the relative proportions of postoperative, inflammatory, vascular and other causes. The absolute case numbers are indicated within the corresponding segments.

A total of 44 patients underwent IFSG transplantation, consisting of 33 males and 11 females, with a mean age of 72.41 ± 11.31 years (median age of 72; age range of 47–91). The treated wounds were notably large, with a median area of 42.5 cm^2^ and a mean area of 84.51 ± 105.66 cm^2^ (Table 1). The majority of the treated wound sites were located on the lower legs, followed by the scalp and face. Table 2 and Figure 2 provide an overview of the various wound types, including acute postoperative wounds following excision of skin cancer and its metastasis (24; 54.54%), as well as chronic inflammatory wounds such as those secondary to pyoderma gangrenosum (6; 13.64%), chronic ulcers of vascular origin (6; 13.64%) such as livedoid vasculopathy (1; 2.27%) and cryoglobulinemic vasculitic ulcers (1; 2.27%) and wounds of other origin (8; 18.18%) such as those stemming from a wound healing disorder (4; 9.09%) and graft‐versus‐host disease of the skin (1; 2.27%).

**TABLE 1:** Patient characteristics.

Characteristics	*n* = 44
Age (years)	
Median (range)	72 (47–91)
Mean (SD)	72.41 (11.31)
Gender, *n* (%)	
Male	33 (75)
Female	11 (25)
Wound area (cm^2^)	
Median (range)	42.5 (6–560)
Mean (SD)	84.51 (105.66)
Wound site, *n* (%)	
Lower leg	21 (47.72)
Scalp	14 (31.82)
Face	7 (15.91)
Foot	1 (2.27)
Upper leg	1 (2.27)

Abbreviation: SD, standard deviation.

**TABLE 2:** Wound types.

Characteristics	*n* = 44
Wound type, *n* (%)	
Postoperative	
Basal cell carcinoma	11 (25)
Squamous cell carcinoma	7 (15.91)
Atypical fibroxanthoma	1 (2.27)
Pleomorphic dermal sarcoma	1 (2.27)
Melanoma metastasis	2 (4.54)
Merkel cell carcinoma	1 (2.27)
Kaposi's sarcoma	1 (2.27)
Inflammatory	
Pyoderma gangrenosum	6 (13.64)
Vascular	6 (13.64)
Martorell's ulcer	2 (4.54)
Livedoid vasculopathy	1 (2.27)
Cryoglobulinemic vasculitis	1 (2.27)
ANCA negative vasculitis	1 (2.27)
Chronic venous ulceration	1 (2.27)
Other	8 (18.18)
Wound healing disorder, not specified	4 (9.09)
Ulcus cruris, others	2 (4.54)
Graft‐versus‐Host‐disease	1 (2.27)
Combustio	1 (2.27)

### 3.2 Transplantation of IFSG Improves Wound Healing

Evaluation of the clinical outcomes of the 44 patients revealed that 39 patients exhibited positive responses to transplantation of IFSG, demonstrating adequate granulation tissue formation (Table 3). Conversely, five patients did not show improvement in wound healing. Of the 39 patients with a positive response to IFSG, 20 patients achieved complete wound healing by secondary intention following IFSG transplantation, and 19 patients exhibited initial partial wound healing. Of these 19 patients, five developed sufficient granulation tissue to undergo subsequent reconstructive procedures and were then able to achieve complete epithelialization. Specifically, two patients received split‐thickness skin grafts, one patient underwent a full‐thickness skin graft, one patient was treated with nasolabial flap reconstruction and one patient received alar reconstruction via a local transposition flap.

**TABLE 3:** Clinical outcome.

	Overall	Chronic	Acute
Outcome	*n* = 44	*n* = 22	*n* = 22
Wound healing, *n* (%)			
Responder	39 (88.63)	17 (77.27)	22 (100)
None responder	5 (11.36)	5 (22.73)	0 (0)
Wound closure, *n* (%)			
Complete	20 (45.45)	9 (40.90)	11 (50)
Partial	19 (43.18)	8 (36.36)	11 (50)
None	5 (11.36)	5 (22.73)	0 (0)

### 3.3 IFSG Promotes Healing Across Multiple Wound Types

**FIGURE 3:**
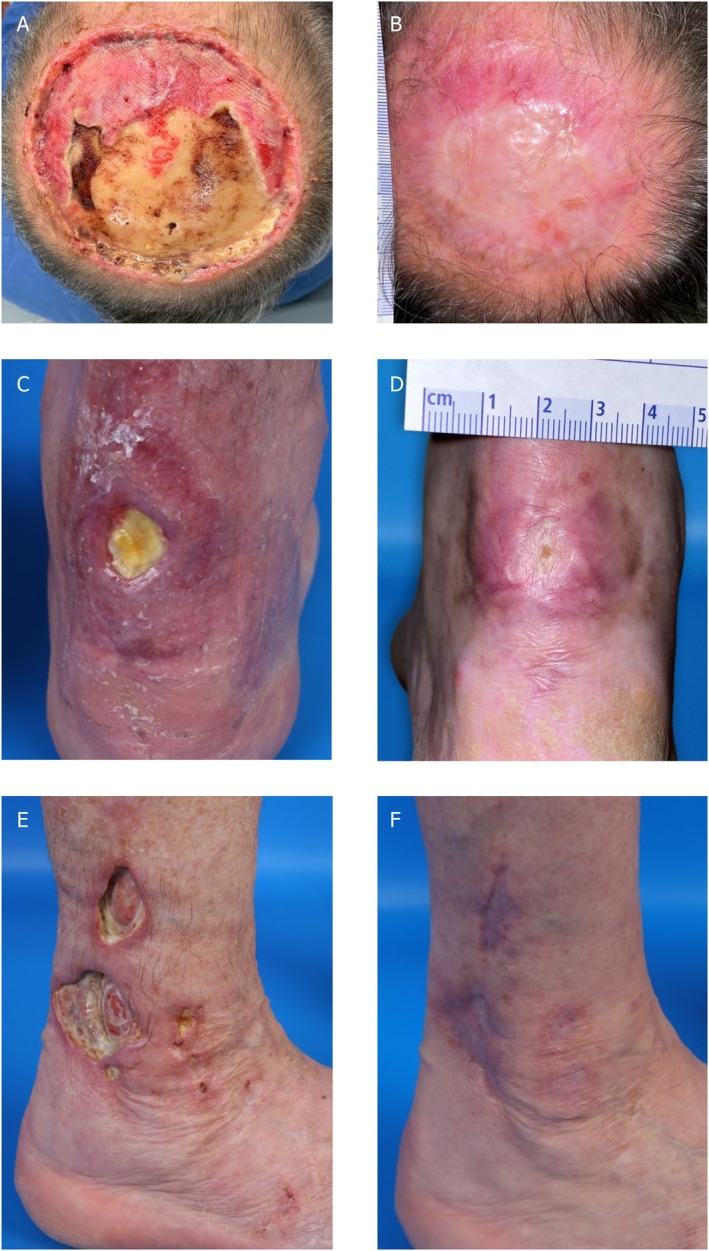
Patient (P02) following excision of squamous cell carcinoma after surgical scalping, with exposed bone at 2 months (A) and complete healing 6 months postoperatively (B). The patient is currently undergoing immunosuppressive therapy with cyclosporine, prednisolone and mycophenolate mofetil after kidney transplantation. Clinical progression of a patient (P27) with a wound healing disorder over a 6‐month period following an Achilles tendon rupture, illustrating the condition prior to transplantation (C) and at 2 months post‐transplantation (D). Notably, fish skin transplantation resulted in a significant reduction in the wound area and complete healing. Patient (P25) with ulcerations due to livedovasculopathy, depicted before transplantation (E) and at 12 months post‐transplantation (F), demonstrating complete healing.

The 44 wounds were categorized into 22 acute and 22 chronic types. Among the acute wounds, all cases (100%) demonstrated clinically significant improvement, with 11 patients achieving complete healing (Figure 3A,B) and 11 experiencing partial wound closure. These acute wounds primarily included substantial defects and/or defects with exposed bone after tumour excision, as well as wounds in challenging locations such as the scalp. Of the 22 chronic wounds, 77.27% showed clinical improvement. Nine patients (40.9%) achieved complete wound healing, whereas eight patients (36.36%) experienced at least partial healing. Five patients with chronic wounds did not derive meaningful benefits from IFSG transplantation. Chronic wounds frequently affected the Achilles tendon region (Figure 3C,D) and often stemmed from wound healing disorders such as hypertension (leading to Martorell ulcers), livedoid vasculopathy (Figure 3E,F) and pyoderma gangrenosum. Non‐healed wounds included those secondary to pyoderma gangrenosum and cryoglobulinemic vasculitis, as well as wounds from treated melanoma metastases. Wounds from pyoderma gangrenosum that did not heal were resistant to therapy despite multiple systemic anti‐inflammatory treatments. However, ulcerations caused by cryoglobulinemic vasculitis showed no deterioration during the observation period. Chronic wounds associated with melanoma metastases were managed with radiation therapy, chemoradiation, and, as part of immunotherapy, additional local IL‐2 injections.

### 3.4 IFSG Appears to Be Safe and Well‐Tolerated

Overall, this procedure appears to be safe. None of the patients reported an allergic reaction to the fish. With the exception of one patient, all participants expressed willingness to undergo transplantation with IFSG again. However, one patient experienced significant pain following the removal of the vacuum dressing due to the adhesive bandage adhering to scalp hair, which led to the decision not to have the transplantation carried out again. Surgery‐associated adverse events such as bleeding, necrosis or local and systemic infections were not observed. According to a written patient survey, 5 out of 20 patients reported experiencing pain ranging from ‘quite a lot’ to ‘severe’ during transplantation, whereas 13 patients reported pain levels from ‘not at all’ to ‘a little’. Notably, in post‐tumour patients, no local tumour recurrence was observed throughout a follow‐up period of up to five years following transplantation with IFSG.

### 3.5 High Patient Satisfaction During and After Transplantation of IFSG

**FIGURE 4:**
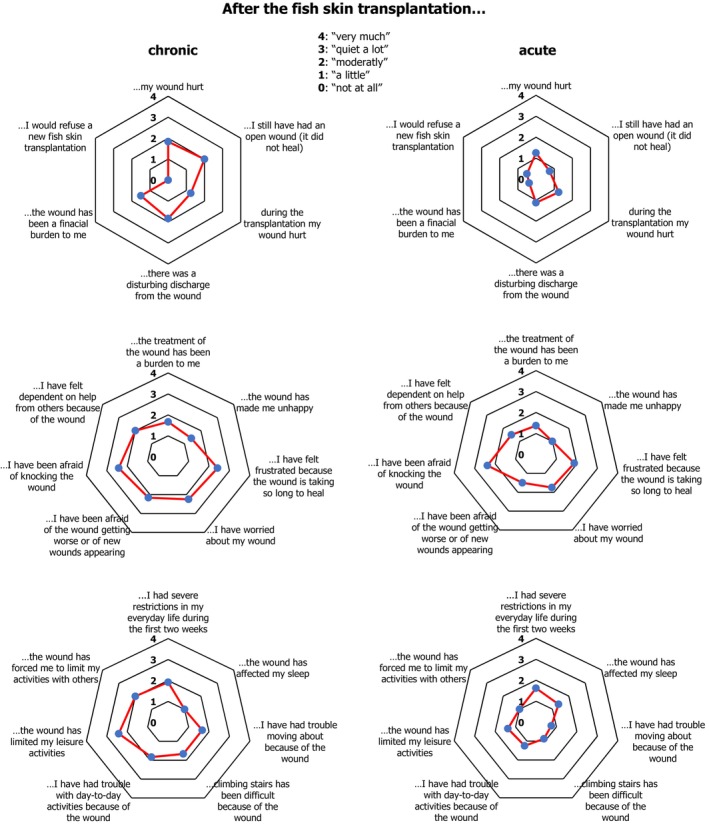
Spider plots showing the mean scores of the adapted Wound‐QoL domains in patients with chronic (left; *n* = 12) and acute (right; *n* = 8) wounds after fish skin transplantation. Each axis represents a statement from the adapted wound QoL questionnaire. Numerical values were assigned to each item response (0 = ‘not at all’ 1 = ‘a little’ 2 = ‘moderately’ 3 = ‘quite a lot’ 4 = ‘very much’), and mean scores were calculated for each domain. Scores are presented in blue.

In response to the feedback gathered from patient experiences, a detailed questionnaire was distributed to explore various facets of the transplantation process. This survey covered topics such as wound healing, recovery, pain intensity, daily life restrictions and willingness to undergo the procedure again. Concerning wound healing, 65% (13 out of 20) of the patients indicated that they had minimal or no residual wounds, while 35% (7 out of 20) reported moderate to significant unhealed wounds. During the operation, 13 out of 20 patients experienced no to minimal pain, whereas two patients reported significant pain (Table 4). Regarding postoperative pain, 11 out of 20 patients experienced either no or minimal pain, while three described their pain as moderate. Six patients reported considerable to severe postoperative pain. After the procedure, 70% of the participants experienced no to moderate limitations in their daily activities, while 30% faced quite a lot to significant restrictions in daily life. Ultimately, a large majority (95%) expressed willingness to undergo future transplantation with IFSG. A comprehensive analysis of the questionnaire responses is presented in Table 4 and Figure 4.

**TABLE 4:** Patient survey (*n* = 20).

After the fish skin transplantation…	Not at all	A little	Moderately	Quiet a lot	Very much
My wound hurt	6	5	3	3	3
I still have had an open wound (it did not heal)	7	5	1	5	2
During the transplantation my wound hurt	9	4	2	3	2
I had severe restrictions in my everyday life during the first two weeks	5	5	4	1	5
There was a disturbing discharge from the wound	5	6	4	3	2
The wound has affected my sleep	8	5	4	2	1
The treatment of the wound has been a burden to me	5	5	6	2	2
The wound has made me unhappy	8	4	4	3	1
I have felt frustrated because the wound is taking so long to heal	2	6	3	4	5
I have worried about my wound	5	3	2	6	4
I have been afraid of the wound getting worse or of new wounds appearing	5	5	2	3	5
I have been afraid of knocking the wound	2	5	1	7	5
I have had trouble moving about because of the wound	8	4	3	4	1
Climbing stairs has been difficult because of the wound	8	4	3	3	2
I have had trouble with day‐to‐day activities because of the wound	5	6	3	4	2
The wound has limited my leisure activities	5	4	2	4	5
The wound has forced me to limit my activities with others	6	5	3	3	3
I have felt dependent on help from others because of the wound	4	8	1	2	5
The wound has been a finacial burden to me	10	6	0	1	3
I would refuse a new fish skin transplantation	19	0	0	0	1

### 3.6 Confocal Microscopy

**FIGURE 5:**
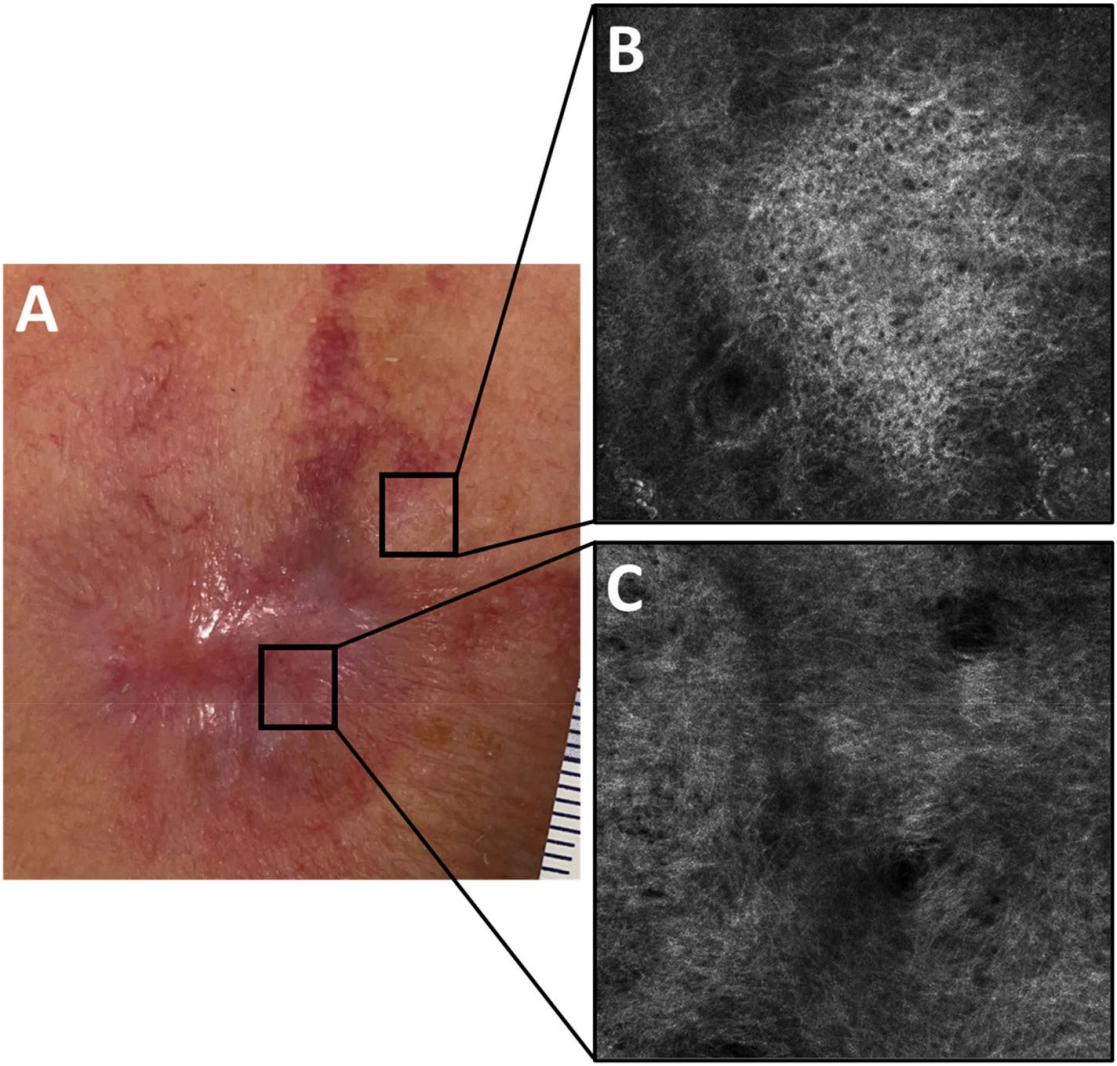
Clinical (A) and confocal microscopy images (B, C) of patient P04 following excision of a recurrent basal cell carcinoma. The black squares delineate the regions of interest for confocal microscopy: The perilesional region (B) adjacent to the transplanted fish skin and the lesional area representing scar tissue (C) subsequent to fish skin transplantation. Both images were obtained within the superficial dermis. In the perilesional region, collagen fibres appeared thicker than in the lesional area, where the fibres were thinner and exhibited a more reticular pattern.

We performed confocal microscopy imaging on 11 patients to examine and compare the morphology and structure of collagen fibres in the superficial dermis between the lesional and perilesional areas. Images were obtained at various intervals during healing. In the majority of images, the collagen fibres in the lesional areas appeared thin and were organized in a net‐like pattern, similar to those in the perilesional areas (Figure 5).

## 4 Discussion

Complex postoperative acute wounds and chronic wounds pose considerable challenges for dermatologists. In addition to established techniques, such as autologous split‐thickness skin grafting, various alternative surgical approaches are being developed to expedite wound closure, particularly when the likelihood of successful healing with autologous split‐thickness skin graft is low, as in the case of a chronic inflammatory wound or a wound with exposed bone [19]. Therefore, it is essential to implement a bridge‐to‐transplant procedure or intervention that facilitates wound healing. Given the existence of various established skin substitute techniques, such as acellular dermal matrices, it is crucial to investigate alternative approaches to enhance wound healing. This study systematically assessed the treatment outcomes of 44 patients managed with the innovative dermal substitute Kerecis IFSG. In almost every case, a single graft application was sufficient, obviating the need for additional closure techniques. The most frequent anatomical site of application was the lower leg, followed by the head region, particularly in cases involving the exposed calvarial bone. Large postoperative wound defects following tumour excision, as well as chronic wounds associated with complex underlying conditions such as pyoderma gangraenosum, were the primary indications for employing IFSG, typically after conventional therapies had failed (Figure 2). It has previously been demonstrated that IFSG can promote wound healing in real world settings; clinical studies and preclinical studies. Accelerated wound healing has been observed in wounds treated with IFSG, including in both preclinical and clinical burn studies; and reviews on burn wound management, accelerated healing has been observed [20, 21]. Moreover, acellular fish skin has been shown to support tissue regeneration in vascular ulcers and diabetic foot ulcers [13, 22, 23, 24]. In a controlled human study using 4 mm punch biopsies to create acute skin injuries, IFSG‐treated wounds achieved faster epithelialization compared to human amnion/chorion membrane allograft‐treated wounds [25].

In our study, we were able to show that even in wounds with exposed bone or tendons, healing could be successfully achieved following a single application of IFSG. Similar results were reported by Dorweiler et al., particularly in cases of wounds exposing bone related to vascular insufficiency or following amputation [13].

To date, no published reports are available describing the use of acellular fish skin in rare ulcerative conditions such as livedoid vasculopathy, graft‐versus‐host diseases, cryoglobulinemic vasculitis or pyoderma gangraenosum. Our findings may therefore provide the first indications of potential applicability in these challenging wound types.

**FIGURE 6:**
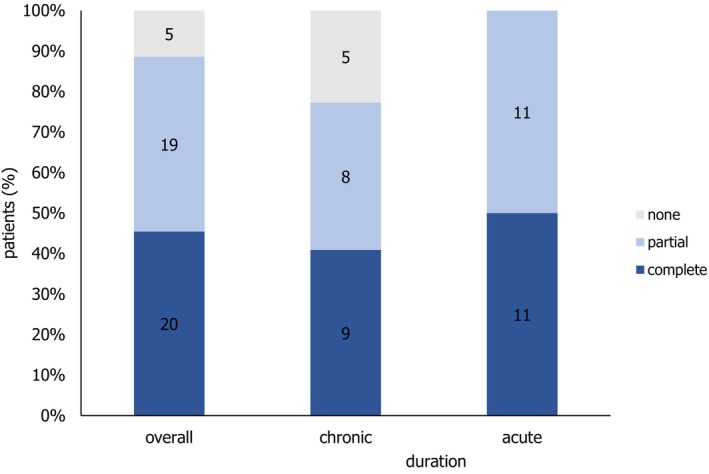
Distribution of epithelialization outcomes (complete, partial and none) in relation to the overall wound cohort, chronic wounds and acute wounds. Values are expressed as absolute number of patients. Percentages refer to the proportion within each group.

Across the cohort of both acute and chronic wounds, an overall response rate of approximately 89% (*n* = 39) was observed, with only five patients classified as non‐responders because of the absence of measurable improvement in wound healing (Table 3 and Figure 6). Notably, all patients with wounds of less than eight weeks' duration exhibited granulation tissue formation and achieved either partial or complete wound closure following treatment with fish skin. Only five patients from the chronic wound subgroup did not demonstrate satisfactory healing progress. One patient underwent multiple excisions of cutaneous metastasis of melanoma on the head and further treatments, such as radiation and local chemotherapy. These wounds did not heal, but there was a clearly restricted wound‐healing environment. In these cases, uncontrolled underlying systemic disease was identified as a significant limiting factor for wound healing and may represent a key reason for the lack of response to transplanted fish skin. Interestingly, in the majority of patients treated with IFSG, complete secondary wound healing was achieved without the need for additional surgical interventions. This finding is particularly relevant for elderly or multimorbid patients, who may benefit not only from the ‘bridge‐to‐transplant’ function of IFSG, but also from the potential of these biologic tissue grafts to support complete wound healing by secondary intention. This outcome was somewhat unexpected, as acellular dermal matrices are typically intended to promote sufficient granulation tissue to prepare the wound bed for subsequent autologous skin grafting procedures [26].

The transplantation procedure is minimally invasive and can be performed on an outpatient basis, resulting in minimal trauma. Our study demonstrated that IFSG transplantation is a rapid and patient‐friendly option to stimulate wound healing, supported by high patient satisfaction, as evidenced by a 95% willingness to undergo the procedure again. In our confocal images, the collagen fibres in the lesional, superficial dermis appeared thin and reticular. Longo et al. described this as indicative of the skin in young patients [27]. Thin parallel‐arranged fibres have also been observed following fractional laser treatment. Thus, findings of the present study indicate that IFSG facilitates wound healing and favours the development of a well‐organized, thin and reticular fibre network similar to young and flexible skin [27]. In addition to the known benefits of IFSG in wound healing, its impact on the wound microbiome and inflammasome remains an understudied but potentially important aspect. Further studies are warranted in this regard.

Our study highlights that the minimally invasive technique of IFSG transplantation is straightforward, adaptable and safe for a diverse array of wound types. IFSG has the potential to prevent the chronification of excisional wounds and serves as a supportive and well‐tolerated treatment in the management of chronic wounds. Further research and larger clinical trials are necessary to further establish the clinical efficacy, safety and contraindications of this therapy.

In conclusion, IFSG transplantation represents a promising and transformative intervention for the management of chronic or hard‐to‐heal wounds of various origins, potentially improving patient outcomes and reducing health care costs.

### 4.1 Limitations

This study has several limitations that should be considered when interpreting the results. First, its retrospective design inherently limits causal inference and may be subject to selection bias. Second, the absence of a control group limits the ability to draw causal conclusions regarding the effectiveness of IFSG. Therefore, the results of this study should be interpreted as descriptive and hypothesis‐generating rather than confirmatory. Third, the relatively small sample size and the heterogeneity of wound etiologies, while reflective of real‐world clinical practice, limit the ability to perform robust subgroup analyses, particularly for rare conditions such as pyoderma gangrenosum or cryoglobulinemic vasculitis.

Additionally, the study population comprises a heterogeneous group of acute and chronic wounds of different etiologies and due to the limited sample size and exploratory design, no statistical adjustment for potential confounders was performed, which limits comparability across subgroups.

Wound healing outcomes were primarily assessed based on clinical evaluation and photographic documentation rather than standardized quantitative wound measurement tools, which may introduce a degree of observer bias. Furthermore, outcome definitions were applied retrospectively and based on non‐blinded clinical and photographic assessments, further increasing the risk of observer bias. Fourth, the Wound‐QoL questionnaire was used in a minor modified, non‐validated form with changes in item selection and the addition of procedure‐specific questions, which may limit the validity and comparability of patient‐reported outcomes.

Nevertheless, the consistent clinical improvement observed across a broad spectrum of complex acute and chronic wounds, together with high patient satisfaction and an excellent safety profile, supports the clinical relevance of our findings. Prospective, controlled studies with larger patient cohorts are warranted to further validate these results and to better define optimal indications and treatment algorithms for IFSG.

## Funding

The authors have nothing to report.

## Ethics Statement

This retrospective study was approved by the Ethics Committee of Heinrich Heine University Düsseldorf (Number 2025–3178, 26.02.2025).

## Conflicts of Interest

The authors declare no conflicts of interest.

## Supporting information


**Table S1:** Detailed patient information, acute wounds. Detailed patient information, chronic wounds.

**Data S1:** iwj70931‐sup‐0002‐Supinfo1.docx.

**Data S2:** iwj70931‐sup‐0003‐Supinfo2.docx.

**Data S3:** iwj70931‐sup‐0004‐Supinfo3.docx.

## Data Availability

All relevant data are presented in the paper and its Supporting Information files. The raw data supporting the conclusions of this study will be made available by the authors upon request.
