# Supplementary material for: Evaluation of Acellular Intact Fish Skin Grafts for Treating Acute and Chronic Wounds
Source: Int Wound J. 2026 Apr 27;23(5):e70931. doi: 10.1111/iwj.70931 (PMC13120852; doi:10.1111/iwj.70931)
Supplement: Supplementary file 1 — Table S1: Detailed patient information, acute wounds. Detailed patient information, chronic wounds. [file IWJ-23-e70931-s003.docx]

| **Supplementary Table 1. Detailed patient information, acute wounds** | | | | |  |  |  |  |
| --- | --- | --- | --- | --- | --- | --- | --- | --- |
| **patient** | **age** | **gender** | **cause of wound** | **localisation** | **wound area (cm²)** | **treatment outcome** | **co-treatments** | **comorbidities** |
| P01 | 81 | m | excision of basal cell carcinoma | left cheek | 49 | complete healing |  |  |
| P02 | 60 | m | excision of squamous cell carcinoma | scalp exposing bone | 182 | complete healing | systemic immunospression | status post living  donor kidney transplantation |
| P03 | 88 | m | excision of basal cell carcinoma | nose exposing bone | 10.50 | complete healing |  |  |
| P04 | 79 | m | excision of a recurrence  of a basal cell carcinoma | left temple | 12 | complete healing |  |  |
| P05 | 80 | m | combustion with  accompanying erysipelas | left lower leg | 140 | complete healing |  | metabolic syndrome,  Parkinson's disease |
| P06 | 68 | m | excision of basal cell carcinoma | left upper leg | 30 | complete healing |  | arterial hypertension |
| P07 | 68 | m | excision of basal cell carcinoma | scalp | 49 | complete healing |  |  |
| P08 | 88 | m | excision of atypical fibroxanthoma | scalp | 20 | complete healing |  |  |
| P09 | 75 | m | excsision of merkel cell carcinoma | scalp exposing bone | 42 | complete healing |  |  |
| P10 | 72 | w | excision of basal cell carcinoma | right ear | 30 | complete healing |  |  |
| P11 | 85 | m | excision of squamous cell carcinoma | scalp exposing bone  (first time granules following fish skin 4 month later) | 81 | complete healing | systemic immunosuppression | type 2 diabetes,  myasthenia gravis |
| P12 | 85 | m | excision of basal cell carcinoma | scalp | 20 | partial healing | split-thickness skin graft,  local radiatio |  |
| P13 | 78 | w | excision of basal cell carcinoma | left lower leg | 60 | partial healing | split-thickness skin graft,  systemic immunospression | metabolic syndrome,  rheumatoid arthritis |
| P14 | 83 | m | excision of squamous cell carcinoma | scalp | 49 | partial healing |  |  |
| P15 | 83 | m | excision of squamous cell carcinoma | scalp exposing bone | 238 | partial healing | hair follicle transplantation |  |
| P16 | 91 | m | excision of squamous cell carcinoma | scalp | 70 | partial healing |  |  |
| P17 | 84 | m | excision of plemorphic dermal sarcoma | scalp exposing bone (granules) | 100 | partial healing | Split-thickness skin graft following  complete granulation to skin level then radiatio | type 2 diabetes |
| P18 | 88 | w | excision of basal cell carcinoma | nose | 12 | partial healing | nasolabial flap reconstruction | type 2 diabetes,  cardiac arrythmia |
| P19 | 64 | w | excision of basal cell carcinoma | left temple | 35 | partial healing | full-thickness skin graft following  complete granulation to skin level |  |
| P20 | 71 | m | excision of squamous cell carcinoma | scalp exposing bone | 70 | partial healing | systemic immunsupression, local radiatio | post-heart transplantation,  type 2 diabetes |
| P21 | 89 | m | excision of squamous cell carcinoma  (non in sano) | scalp exposing bone | 120 | partial healing |  | dementia |
| P22 | 85 | m | excision of basal cell carcinoma | nasal dorsum | 12 | partial healing  before flap | Alar reconstruction via  local transposition flap | type 2 diabetes |
|  |  |  |  |  |  |  |  |  |
| **Supplementary Table 1. Detailed patient information, chronic wounds** | | | | | | | | |
| **patient** | **age** | **gender** | **cause of wound** | **localisation** | **wound area (cm²)** | **treatment outcome** | **co-treatments** | **comorbidities** |
| P23 | 62 | m | wound healing disorder | right lower leg  exposing achilles tendon | 21 | complete healing | none | type 2 diabetes |
| P24 | 62 | w | pyoderma gangrenosum | left lower leg | 80 | complete healing | systemic immunosuppression, hyperbaric oxygen therapy,  intravenous immunoglobuline, hair follicle transplantation | metabolic syndrome |
| P25 | 52 | w | livedoid vasculopathy | right lower leg | 70 | complete healing | iloprost, rivaroxaban |  |
| P26 | 76 | m | Martorell`s ulcer | right lower leg | 21 | complete healing |  | metabolic syndrome |
| P27 | 61 | m | wound healing disorder | right lower leg achilles tendon | 10,50 | complete healing |  |  |
| P28 | 81 | w | Martorell`s ulcer | left lower leg | 120 | complete healing |  | type 2 diabetes,  metabolic syndrome |
| P29 | 74 | w | wound healing disorder after excision  of dermatofibrosarcoma protuberans | left lower leg | 36 | complete healing |  | type 2 diabetes,  metabolic syndrome |
| P30 | 69 | m | Graft-versus-Host-disease | right lower leg | lateral: 70 medial:14  ventral: 9 | complete healing | Immunsupression,  extracorporal photopheresis | allogenic stem cell transplantation |
| P31 | 70 | m | pyoderma gangrenosum and vasculopathy | left foot  exposing tendon | 21 | complete healing | systemic immunosupression |  |
| P32 | 82 | m | melanoma metastasis | scalp | 12 | partial healing | local radiatio, intralesional Interleukin-2-injection, systemic PD-1 Inhibition, |  |
| P33 | 70 | m | pyoderma gangrenosum | right lower leg | 238 | partial healing | systemic immunosuppression, intravenous immunoglobuline, hair follicle transplantation | metabolic syndrome |
| P34 | 63 | m | wound healing disorder after  excision of squamous cell carcinoma | scalp  exposing bone | 11 | partial healing | systemic immunsupression, local radiatio | post-heart transplantation,  type 2 diabetes |
| P35 | 78 | m | cryoglobulinemic vasculitis | left lower leg | 300 | partial healing |  |  |
| P36 | 47 | w | ulcera crura | lower leg | left 560; right:84 | partial healing | systemic immunsupression |  |
| P37 | 57 | m | pyoderma gangrenosum | both lower legs | 4 on the left &  one on the right; overall: 43.5 | partial healing |  |  |
| P38 | 72 | m | chronic venous ulceration | left lower leg | 6 and 24 | partial healing |  |  |
| P39 | 63 | m | mixed ulcus cruris | left lower leg | 25 | partial healing | compression | Faktor V Leiden,  peripheral artery disease,  post- thrombotic syndrome |
| P40 | 59 | w | pyoderma gangrenosum | right lower leg  exposing tendon | 240 | no healing | systemic immunosuppression, intravenous immunoglobuline,  hair follicle transplantation | colitis ulcerosa |
| P41 | 55 | m | cutaneous melanoma metastasis | right knee | 36 | no healing | intralesional Interleukin-2-injection, local electrochemotherapy, systemic PD-1Inhibition, |  |
| P42 | 63 | m | Kaposi’s sarcoma | left lower leg | 170 | no healing |  |  |
| P43 | 59 | m | pyoderma gangrenosum | right lower leg | 63 | no healing | Anti-IL17, Anti IL-23,  intravenous immunoglobuline, prednisolone | metabolic syndrome, chronic venous insufficiency |
| P44 | 67 | w | ANCA negative vasculitis | left lower leg | 340 | no healing | systemic immunosuppression, intravenous immunoglobuline |  |
