# Supplementary material for: Evaluation of Acellular Intact Fish Skin Grafts for Treating Acute and Chronic Wounds
Source: Int Wound J. 2026 Apr 27;23(5):e70931. doi: 10.1111/iwj.70931 (PMC13120852; doi:10.1111/iwj.70931)
Supplement: Supplementary file 2 — Data S1: iwj70931‐sup‐0002‐Supinfo1.docx. [file IWJ-23-e70931-s002.docx]

**Patientenfragebogen**

orientiert an „Wound-QoL" Fragebogen zur Lebensqualität bei chronischen Wunden, Augustin *et al*. 2014, Blome *et al.* 2014

**Unterschrift Patienten/-in**

_______________________________

(Name und Vorname in Druckschrift)

_______________________________ ________________________

(Datum) (Unterschrift)

|  | Nach der Fischhauttransplantation … | gar nicht | etwas | mittelmäßig | ziemlich | sehr |
| --- | --- | --- | --- | --- | --- | --- |
| 1 | …hatte ich Schmerzen an der Wunde |  |  |  |  |  |
| 2 | … hatte ich weiterhin eine offene Wunde (kam es nicht zur Abheilung) |  |  |  |  |  |
| 3 | Während der Transplantation hatte ich Schmerzen an der Wunde |  |  |  |  |  |
| 4 | ...hatte ich in den ersten zwei Wochen starke Einschränkungen in meinem Alltag |  |  |  |  |  |
| 5 | …hatte ich störenden Wundausfluss |  |  |  |  |  |
| 6 | …war mein Schlaf durch die Wunde beeinträchtigt |  |  |  |  |  |
| 7 | …war die Behandlung der Wunde für mich belastend |  |  |  |  |  |
| 8 | …war ich wegen der Wunde niedergeschlagen |  |  |  |  |  |
| 9 | …hat es mich frustriert, dass die Heilung so lange dauert |  |  |  |  |  |
| 10 | …habe ich mir Sorgen wegen meiner Wunde gemacht |  |  |  |  |  |
| 11 | …hatte ich Angst vor einer Verschlechterung oder vor neuen Wunden |  |  |  |  |  |
| 12 | …hatte ich Angst, mich an der Wunde zu stoßen |  |  |  |  |  |
| 13 | …konnte ich mich wegen der Wunde schlecht fortbewegen |  |  |  |  |  |
| 14 | …war das Treppensteigen wegen der Wunde mühsam |  |  |  |  |  |
| 15 | …hatte ich wegen der Wunde Probleme mit Alltagstätigkeiten |  |  |  |  |  |
| 16 | …waren meine Freizeitaktivitäten wegen der Wunde eingeschränkt |  |  |  |  |  |
| 17 | …musste ich wegen der Wunde Aktivitäten mit Anderen einschränken |  |  |  |  |  |
| 18 | …fühlte ich mich wegen der Wunde abhängig von der Hilfe Anderer |  |  |  |  |  |
| 19 | …war die Wunde für mich eine finanzielle Belastung |  |  |  |  |  |
| 20 | … würde ich eine erneute Fischhauttransplantation ablehnen |  |  |  |  |  |
