# Supplementary material for: Evaluation of Acellular Intact Fish Skin Grafts for Treating Acute and Chronic Wounds
Source: Int Wound J. 2026 Apr 27;23(5):e70931. doi: 10.1111/iwj.70931 (PMC13120852; doi:10.1111/iwj.70931)
Supplement: Supplementary file 4 — Data S3: iwj70931‐sup‐0004‐Supinfo3.docx. [file IWJ-23-e70931-s001.docx]

**Patient questionnaire**

based on the “Wound-QoL” questionnaire on quality of life in chronic wounds (Augustin et al. 2014; Blome et al. 2014)

**patient´s signature**

_______________________________

(surname and first name in block letters )

_______________________________ ________________________

(date) (signature)

|  | After the fish skin transplantation… | not at all | a little | moderately | quiet a lot | very much |
| --- | --- | --- | --- | --- | --- | --- |
| 1 | …my wound hurt |  |  |  |  |  |
| 2 | …I still have had an open wound (it did not heal) |  |  |  |  |  |
| 3 | during the transplantation my wound hurt |  |  |  |  |  |
| 4 | ...I had severe restrictions in my everyday life during the first two weeks |  |  |  |  |  |
| 5 | …there was a disturbing discharge from the wound |  |  |  |  |  |
| 6 | …the wound has affected my sleep |  |  |  |  |  |
| 7 | …the treatment of the wound has been a burden to me |  |  |  |  |  |
| 8 | …the wound has made me unhappy |  |  |  |  |  |
| 9 | …I have felt frustrated because the wound is taking so long to heal |  |  |  |  |  |
| 10 | …I have worried about my wound |  |  |  |  |  |
| 11 | …I have been afraid of the wound getting worse or of new wounds appearing |  |  |  |  |  |
| 12 | …I have been afraid of knocking the wound |  |  |  |  |  |
| 13 | …I have had trouble moving about because of the wound |  |  |  |  |  |
| 14 | …climbing stairs has been difficult because of the wound |  |  |  |  |  |
| 15 | …I have had trouble with day-to-day activities because of the wound |  |  |  |  |  |
| 16 | …the wound has limited my leisure activities |  |  |  |  |  |
| 17 | …the wound has forced me to limit my activities with others |  |  |  |  |  |
| 18 | …I have felt dependent on help from others because of the wound |  |  |  |  |  |
| 19 | …the wound has been a finacial burden to me |  |  |  |  |  |
| 20 | …I would refuse a new fish skin transplantation |  |  |  |  |  |
